# The Relationship Between Circulating Growth Differentiation Factor 15 Levels and Diabetic Retinopathy in Patients With Type 2 Diabetes

**DOI:** 10.3389/fendo.2021.627395

**Published:** 2021-03-15

**Authors:** Yixin Niu, Weiwei Zhang, Jie Shi, Yueming Liu, Hongmei Zhang, Ning Lin, Xiaoyong Li, Li Qin, Zhen Yang, Qing Su

**Affiliations:** Department of Endocrinology, Xinhua Hospital, Shanghai Jiaotong University School of Medicine, Shanghai, China

**Keywords:** GDF-15, type 2 diabetes, mild non-proliferative DR, moderate NPDR, vision-threatening DR

## Abstract

**Objective:**

Growth differentiation factor 15 (GDF-15) is a member of the TGF-*β* superfamily that has anti-inflammatory properties. The objective of this study was to evaluate the relationship between circulating GDF-15 levels and diabetic retinopathy (DR) in patients with type 2 diabetes.

**Materials/Methods:**

A case–control study was performed in which 402 patients with type 2 diabetes were enrolled. Of these, 171 patients had DR and the remaining 231 patients without DR acted as controls. The plasma GDF-15 levels were measured using ELISA, while DR was diagnosed using the canon ophthalmic digital imaging system and the Canon EOS 10D digital camera (Canon, Tokyo, Japan) through a non-pharmacologically dilated pupil.

**Results:**

The levels of GDF-15 were significantly higher in patients with DR [168.9 (112.9–228.3) pg/ml *vs*. 127.8 (96.1–202.8) pg/ml, P < 0.001] compared to controls. Results of the Spearman correlation analysis showed that the GDF-15 levels were positively associated with the duration of diabetes morbidity, fasting plasma glucose, systolic blood pressure, albumin/creatinine ratio, creatinine, and liver enzymes, but negatively associated with eGFR (both P < 0.001). The participants in the highest GDF-15 quartile had a significantly increased risk for DR (OR = 2.15, 95% CI 1.53–3.02) after adjusting for potential cofounders.

**Conclusions:**

The circulating GDF-15 levels are positively associated with DR independent of potential cofounders.

## Introduction

The prevalence of diabetes in China has become a major public health concern with ~9.7% of all adults affected (1). Diabetic retinopathy (DR) is a microvascular complication of diabetes and is the leading cause of blindness among adults of working-age around the world (2). Although DR is initially asymptomatic, its progression is characterized by damage to the retinal microvasculature due to inflammation and oxidative stress caused by chronic hyperglycemia. Recently, studies have focused on inflammatory biomarkers and risk factors for endothelial dysfunction, such as C-reactive protein (CRP), and tumor necrosis factor-α (TNF-α) that are considered to be prognostic factors for the development of DR. These studies have also shown that some cytokines may be associated with the development of DR (3–5).

Growth differentiation factor 15 (GDF-15), also known as macrophage inhibitory cytokine-1(MIC-1) (6), placental transformation growth factor-b (PTGF-b) (7–9), prostate derived factor (PDF) (10), placental bone morphogenetic protein (PLAB) (11, 12), NSAID activated gene-1 (NAG-1) (13, 14), and PL74 (15), are divergent members of the transforming growth factor-*β* (TGF-*β*) superfamily with anti-inflammatory properties. GDF-15 is highly expressed in cardiomyocytes, adipocytes, macrophages, endothelial cells, and vascular smooth muscle cells during tissue injury and inflammatory states. It plays a crucial role in the development and progression of cardiovascular diseases such as heart failure, coronary artery diseases, atrial fibrillation, diabetes, cancer, and cognitive impairment (16, 17). In type 2 diabetes, GDF-15 predicts the development of proteinuria in patients with diabetic nephropathy, suggesting that GDF-15 may be a part of an anti-inflammatory response to microvascular damages (18). Moreover, GDF-15 is associated with a number of circulating proangiogenic endothelial progenitor cells in patients with type 2 diabetes (19). Furthermore, GDF-15 expression is markedly increased before the onset of type 2 diabetes (20), which suggests that GDF-15 is a potential biomarker of DR (21).

Therefore, the current study aimed to investigate the relationship between plasma GDF-15 levels and the risk of DR in a large cohort of type 2 diabetic patients.

## Methods

### Study Population

This was a cohort study aimed at assessing the risk factors associated with the development of diabetic complications. Study participants were type 2 diabetes patients recruited from the Department of Endocrinology at Xinhua Hospital Affiliated to Shanghai Jiaotong University School of Medicine between 2013 and 2014. Diabetes was defined according to the 2008 American Diabetes Association diagnostic criteria (22).

Individuals with the following conditions were excluded from the study: cancer, acquired immune deficiency syndrome, severe psychological disorders, clinical signs or symptoms of inborn errors of metabolism, a history of vitreal surgery, senile dementia, tuberculosis, a cataract on examination, or any other communicable disease. A total of 402 participants with type 2 diabetes were included in this study. Written informed consent was obtained from all participants. The Ethics Committee of Xinhua Hospital Affiliated to Shanghai Jiaotong University School of Medicine approved this study.

### Clinical Data Collection and Biochemical Measurements

Anthropometric measurements, questionnaire, physical examination, and laboratory measurements were performed. The waist circumference was defined as the midway level between the costal margins and the iliac crests. Blood pressure was assessed twice on the right arm after a 15-min rest in a sitting position using a standard mercury sphygmomanometer. BMI was calculated as the weight in kilograms divided by the square height in meters (kg/m^2^). Age, alcohol consumption (yes/no, history of alcohol consumption was defined as “yes”, lack of history was defined as “no”), smoking (yes/no, history of smoking was defined as “yes”; lack of history was defined as “no”), education status, and duration of diabetes morbidity were assessed using interviews. Based on the International Physical Activity Questionnaire scoring protocol, the physical activity level was classified as low, moderate, or high level.

The A1c was measured using high-performance liquid chromatography (BIO-RAD, D10, CA), while fasting plasma glucose levels were tested using the glucose oxidase method (ADVIA-1650 Chemistry System, Bayer, Leverkusen, Germany). Lipid profiles, liver enzyme profiles, and creatinine (Cr) were determined using a Hitachi 7080 analyzer (Hitachi 7080; Tokyo, Japan), while fasting insulin was determined using the radioimmunoassay method (Linco Research, St. Charles, MO). The albumin/creatinine ratio (ACR) was calculated as milligrams of urinary albumin excretion per gram of urinary creatinine. Glomerular filtration rate (eGFR) was estimated using a simplified Modification of Diet in Renal Disease formula recalibrated for the Chinese: 186 × (serum creatinine × 0.011)^−1.154^ × (age)^−0.203^ × (0.742 if female) × 1.233 (23). The homoeostasis model assessment (HOMA) value for insulin resistance (HOMA-IR) was evaluated with the following formula: fasting insulin × fasting glucose/22.5. The HOMA-*β* was calculated using the formula described by Matthews et al. (24).

### Circulating GDF-15 Levels, Adiponectin and CRP

The circulating levels of GDF-15 were determined in duplicate using the Duoset kit for ELISA (DY805; R&D Systems, Minneapolis, MN) according to the instructions of the manufacturer. The ELISA system had an intra-assay coefficient of variation of 3 to 9% and an inter-assay coefficient of variation of 4 to 10.2%.

The circulating adiponectin and C-reactive protein (CRP) were measured using ELISA kits (DY1065 and DY1707, respectively; R&D Systems, Minneapolis, MN) according to the instructions of the manufacturer.

### Diagnosis of Diabetic Retinopathy

Dilated ophthalmic eye examinations including fundus photography were performed by an experienced ophthalmologist. DR was classified as cases without diabetic retinopathy (non-DR), mild non-proliferative DR (NPDR), moderate NPDR and vision-threatening DR (VTDR).

### Assessment of Diabetic Retinopathy

Fundus photography was performed using digital non-mydriatic camera (CR6-45NM; Canon, Lake Success, NY) according to the International Classification of Diabetic Retinopathy (25). The severity of DR was classified as 1) non-DR; 2) mild non-proliferative DR (NPDR); 3) moderate NPDR; 4) severe NPDR; and 5) proliferative DR (PDR). Due to the limited number of study participants with PDR (n = 2), PDR cases were combined with severe NPDR cases to give the vision-threatening DR (VTDR) group. When binocular DR was present and unequal, we used the more advanced DR measurement for analyses. Patients with ungradable retinal fundus photographs of both eyes were excluded from the study.

### Statistical Analysis

Continuous variables with normal distribution were shown as means with SDs, whereas variables with skewed distribution were presented as median with interquartile range. For comparisons between groups, continuous variables were compared using Student *t* tests or Mann–Whitney U tests. Categorical variables were expressed as proportions and compared across groups using *X* ^2^ tests. Spearman correlation analysis was used to calculate the correlation coefficients between GDF-15 and metabolic parameters. Multivariate logistic regression models were performed to determine the potential relationship between GDF-15 levels and the risk of DR. To minimize the potential confounding factors, covariates were selected based on biologic interest, well established risk factors for DR, or associated exposures and outcomes. Variables showing P < 0.05 in the univariable regression were entered into the multivariable model. All statistical analyses were performed with the SPSS software (version 25.0). A two-sided P < 0.05 was considered to be statistically significant.

## Results

### Baseline Characteristics

**Figure 1:**
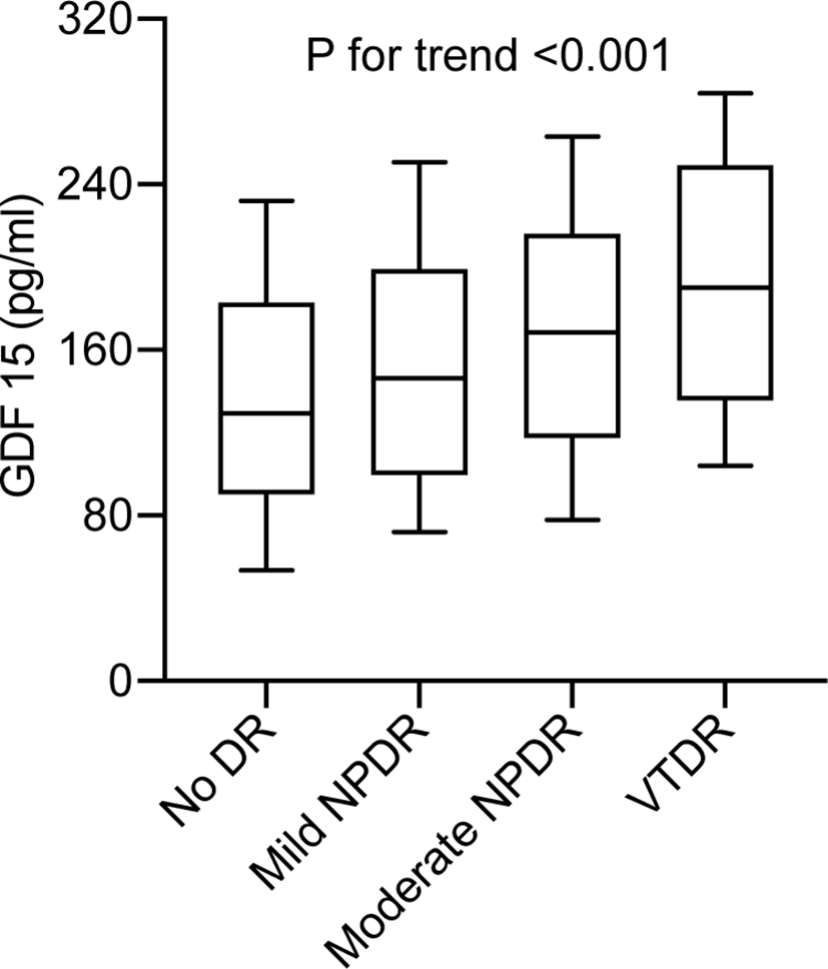
Plasma GDF-15 levels in type 2 diabetes without DR compared with patients with mild non-proliferative DR (NPDR), moderate NPDR, or vision-threatening DR (VTDR). Boxes represent the inter-quartile range, with the median superimposed as a horizontal line. Error bars indicate ranges of plasma GDF-15 levels. *P* < 0.001 for trend.

Out of a study population of 402 participants, 171 individuals (42.5%) had DR. The plasma GDF-15 levels were significantly higher in patients with DR (168.9 [112.9–228.3] pg/ml *vs*. 127.8 [96.1–202.8] pg/ml, P < 0.001) compared to controls. Moreover, there was an increasing trend in the median (inter-quartile range) of GDF-15 concentrations from the patients with no DR, mild NPDR, moderate NPDR, to VTDR (P < 0.001 for trend) (**Figure 1**). The clinical characteristics of the study participants are shown in **Table 1**.

**Table 1:** Characteristics of subjects according to the presence or absence of diabetic retinopathy (DR) (n = 402).

Characteristics	NDR (n = 231)	DR (n = 171)	P value
Case (male/female)	119/112	91/80	0.736
Age (year)	57.8 ± 8.3	57.3 ± 9.2	0.569
Duration (year)	5 (1–10)	10 (7–14)	<0.001
BMI (kg/m^2^)	25.6 ± 3.8	25.3 ± 3.6	0.424
WC (cm)	86.5 ± 8.6	86.9 ± 8.7	0.647
SBP (mmHg)	127.1 ± 11.9	134.5 ± 12.5	<0.001
DBP (mmHg)	75.6 ± 9.2	78.7 ± 10.1	0.002
FPG (mmol/L)	7.6 (5.8–9.7)	9.4 (7.0–11.9)	<0.001
HbA1c (%)	9.1 ± 1.3	9.6 ± 1.5	<0.001
HOMA-IR	2.69 (1.79–3.89)	2.87 (1.85–3.99)	<0.001
TC (mmol/L)	4.83 ± 1.25	4.92 ± 1.31	0.485
TG (mmol/L)	1.68 (1.19–2.53)	1.77 (1.28–2.87)	<0.001
HDL (mmol/L)	1.39 (1.21–1.59)	1.32 (1.16–1.49)	0.009
LDL (mmol/L)	2.91 ± 0.94	2.98 ± 0.95	0.463
CRP (mg/L)	1.92 (1.26–2.70)	2.46 (1.38–3.61)	<0.001
Adiponectin (mg/L)	3.13 (2.39–3.95)	2.87 (2.06–3.73)	0.008
ALT (U/L)	16 (12–25)	22 (14–31)	<0.001
AST (U/L)	20 (16–25)	24 (19–31)	<0.001
GGT (U/L)	22 (16–28)	28 (19–39)	<0.001
Cr (μmol/L)	68.7 ± 18.6	77.9 ± 19.3	<0.001
ACR	10.1 ± 3.6	17.3 ± 3.9	<0.001
eGFR (mL/min/1.73 m^2^)	104.3 (99.9-110.1)	93.6 (88.9–100.3)	<0.001
Hypoglycemic treatments			0.006
Insulin (%)	87 (37.7)	64 (37.4)	
OHA (%)	112 (48.5)	63 (36.8)	
Insulin + OHA (%)	32 (13.9)	44 (25.7)	
GDF-15 (pg/ml)	127.8 (96.1–202.8)	168.9 (112.9–228.3)	<0.001

ACR, albumin/creatinine ratio; ALT, alanine aminotransferase; AST, aspartate transaminase; BMI, body mass index; CRP, C-reactive protein; Cr, creatinine; DBP, diastolic blood pressure; eGFR, estimated glomerular filtration rate; FPG, fasting plasma glucose; GDF15, growth differentiation factor 15; GGT, γ-glutamyltransferase; HbA1c, glycated hemoglobin; HDL-C, high-density lipoprotein cholesterol; HOMA-IR, homeostasis model assessment-insulin resistance; LDL-C, low-density lipoprotein cholesterol; OHA, oral hypoglycemic agent; SBP, systolic blood pressure; TC, total cholesterol; TG, triglycerides; WC, waist circumference.

**Figure 2:**
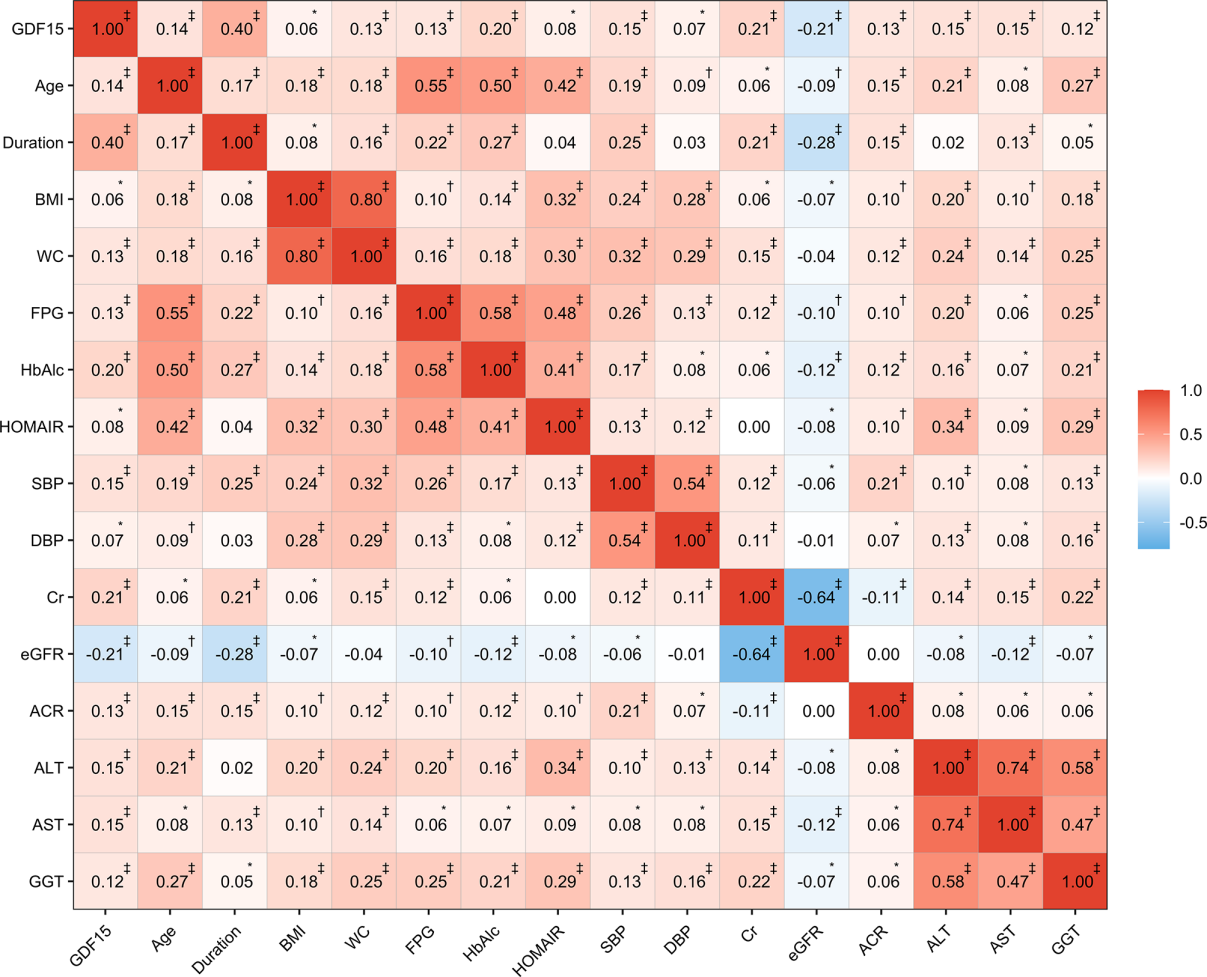
Correlations between GDF-15 and clinical characteristics. The values in the cell represent the correlation coefficients based on spearman correlation. *P < 0.05; ^†^P < 0.001; ^‡^P < 0.0001.

Results of the Spearman correlation analysis showed that GDF-15 was positively correlated with age (r = 0.14, P < 0.0001), duration of morbidity (r = 0.40, P < 0.0001), fasting plasma glucose (r = 0.13, P < 0.0001), systolic blood pressure (r = 0.15, P < 0.0001), Cr (r = 0.21, P < 0.0001), and ACR (r = 0.13, P < 0.0001), but was negatively correlated with eGFR (r = −0.21, P < 0.0001) (**Figure 2**). Multiple regression analysis with a stepwise model was used to assess the independent variables that affect the GDF-15 plasma levels. The variables entered in the model were as follows: age, gender, CRP, BMI, WC, SBP, DBP, ALT, AST, GGT, HbA1c, fasting plasma glucose, fasting plasma insulin, triglycerides, total cholesterol, LDL-c and HDL-c. The main determinants of GDF-15 were age (*β* = 0.304, P < 0.001), ALT (*β* = 0.153, P < 0.001) and Cr (*β* = 0.152, P < 0.001).

### Association Between GDF-15 and DR

**Figure 3:**
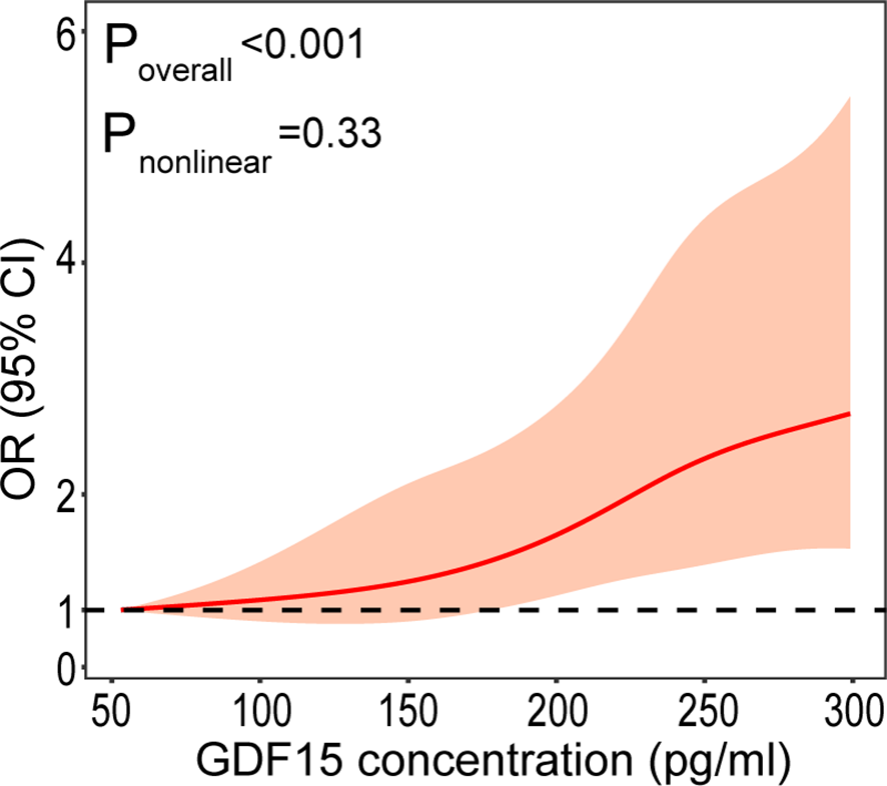
Plasma GDF-15 levels on a continuous scale and the presence of DR. The solid line represents the odds ratio (OR) and the gray area represents the 95% confidence interval (CI). Model was adjusted for age, gender, smoking, drinking, physical activity, educational attainment, BMI, waist circumference, CRP, adiponectin, HOMA-IR, liver enzymes, duration, FPG, eGFR, Cr, and ACR.

**Table 2** displays the odds ratios (ORs) for DR based on the GDF-15 quartiles. As expected, there was an increase in the ORs for DR from the 1st to the 4th GDF-15 quartiles in the study cohort (P < 0.001 for trend). In the highest GDF-15 quartile, the adjusted OR of DR was 2.15 [95% confidence interval (CI) 1.53–3.02] after adjusting for age, gender, smoking, alcohol consumption, education status, physical activity, BMI, waist circumference, CRP, adiponectin, HOMA-IR, liver enzymes, diabetes duration, FPG, eGFR, Cr, and ACR. A positive linear dose–response relationship was evident in the cubic spline regression model (**Figure 3**, P for non-linearity > 0.1).

**Table 2:** Adjusted odds ratios (ORs) of diabetic retinopathy according to quartiles of plasma growth differentiation factor 15 (GDF-15) levels.

	GDF15				P for trend
	Q1	Q2	Q3	Q4	
	n = 100	n = 101	n = 101	n = 100	
Model 1 OR (95% CI)	1.00	1.54 (1.16–2.03)	1.72 (1.31–2.26)	2.61 (1.99–3.40)	<0.001
Model 2 OR (95% CI)	1.00	1.50 (1.13–1.98)	1.64 (1.25–2.17)	2.30 (1.75–3.01)	<0.001
Model 3 OR (95% CI)	1.00	1.47 (1.11–1.95)	1.59 (1.21–2.10)	2.20 (1.67–2.89)	<0.001
Model 4 OR (95% CI)	1.00	1.46 (1.08–1.98)	1.58 (1.15–2.16)	2.15 (1.53–3.02)	<0.001

Model 1 was adjusted for age, gender, smoking, alcohol drinking, educational attainment, and physical activity. Model 2 was further adjusted for BMI and waist circumference. Model 3 was further adjusted for CRP, adiponectin, HOMA-IR, and liver enzymes. Model 4 was further adjusted for duration, FPG, eGFR, Cr, and ACR. Q, quartile. The quartile ranges of Q1, Q2, Q3, and Q4 of plasma GDF-15 level were <96.5, 96.5–141.7, 141.8–210.3, and >210.3 pg/ml, respectively. Q1 is the reference group. Multivariate logistic regression models were used to estimate the odds ratios (ORs) with corresponding 95% confidence intervals (CIs) for diabetic retinopathy.

### Subgroup Analyses

**Figure 4:**
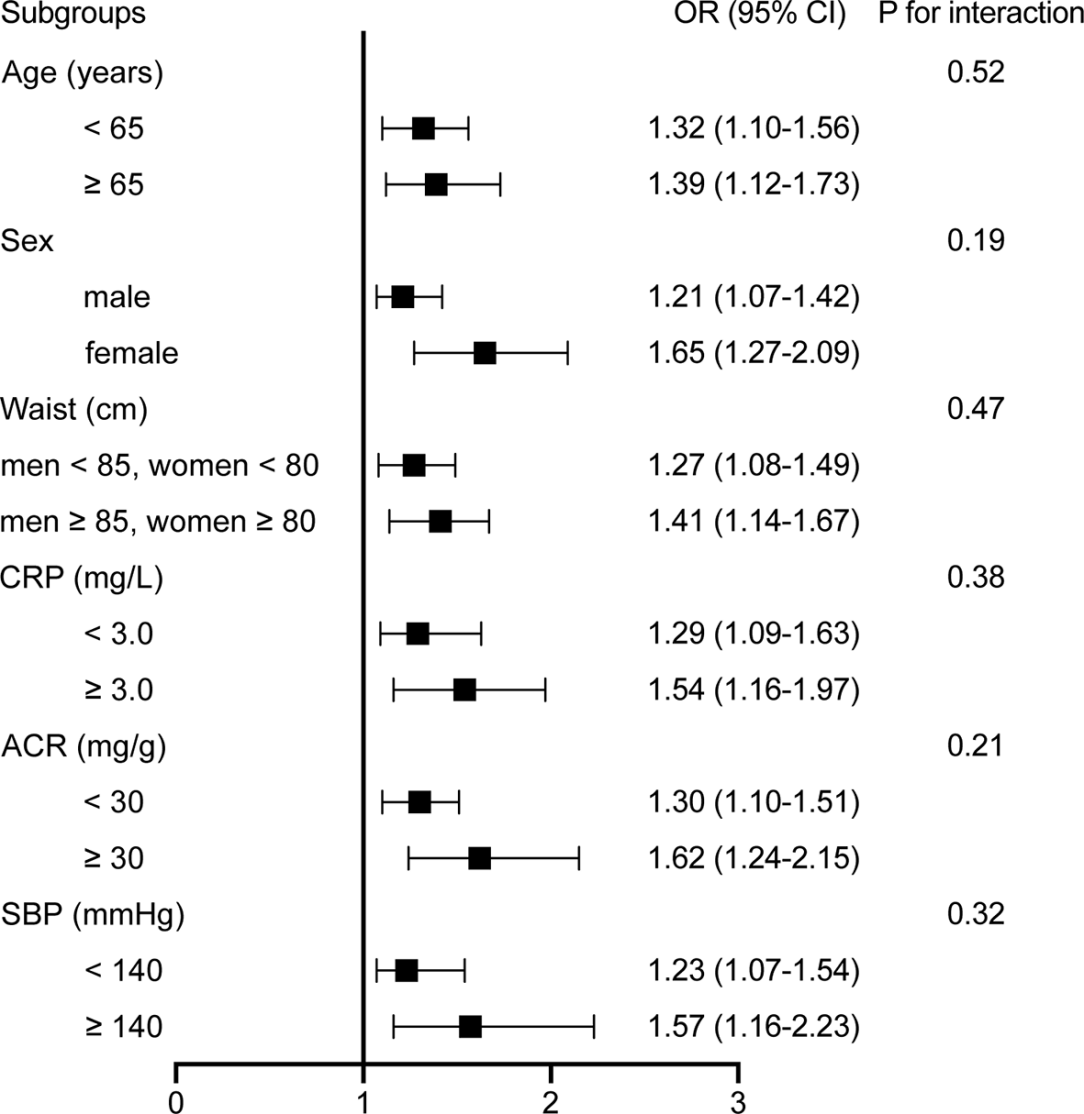
Stratified analyses of the associations [odds ratio (95% confidence interval)] between plasma GDF-15 levels (per 50 pg/ml increment) and DR. Model was adjusted for age, gender, smoking, drinking, physical activity, education status, BMI, waist circumference, CRP, adiponectin, HOMA-IR, liver enzymes, duration, FPG, eGFR, Cr, and ACR. Subgroup variable was excluded from the model.

Subgroup analyses were performed to examine potential effect modifiers, stratified by age (<65 *versus* ≥65 years), sex (male *versus* female), SBP (<140 *versus* ≥140 mmHg), waist circumference (<90 *versus* ≥90 cm for men and <85 *versus* ≥85 cm for women and), CRP (<3.0 *versus* ≥3.0 mg/L), and ACR (<30 *versus* ≥30 mg/g). Results of stratified analyses showed that the positive associations between GDF-15 levels and the presence of DR remained consistent across all subgroups (**Figure 4**). No interaction was observed with any of the variables (all P for interaction > 0.1).

## Discussion

In this cohort study, we found a significantly positive association between circulating GDF-15 levels and the risk of DR in individuals with type 2 diabetes. This association remained even after extensively adjusting for potential confounders through the stratification of several potential risk factors that may have an effect on the GDF-15–DR relationship.

In our study, plasma levels of GDF-15 were significantly higher in patients with DR compared to patients without DR. Moreover, results of our study showed that plasma GDF-15 concentrations were associated with progression of DR in individuals with type 2 diabetes, after controlling for confounding risk factors. Glycemia, blood pressure, and duration of diabetes morbidity have been identified as risk factors for DR although there are still some controversies (26, 27). This is consistent with the findings from our study that showed that the duration of diabetes morbidity, HbA1c, CRP, and systolic blood pressure was a risk factor for DR in patients with type 2 diabetes. In line with a previous study (28), we also found that the plasma levels of GDF-15 were significantly associated with renal damage and could predict the development of diabetic retinopathy in patients with type 2 diabetes. Importantly, the association between circulating plasma GDF-15 concentrations and the progression of DR in type 2 diabetic patients in our study was independent of the traditional DR risk factors mentioned above.

GDF-15 was discovered and cloned as a divergent member of the TGF-*β* superfamily in the late 1990s (6). The GDF-15 gene is composed of two exons and contains one single intron that interrupts the coding sequences at identical positions within the pre-pro-domain of the corresponding proteins (7, 29). It is widely expressed in almost all tissues indicating that it has numerous vital cellular functions such as proliferation, migration, maintenance, and homeostasis (10). This suggests that alterations of serum GDF-15 levels may be associated with various diseases including heart failure, coronary artery diseases, cancer, diabetes, and diabetic renal damage (16–18). Circulating GDF-15 is likely to be secreted by endothelial cells such that the serum levels of GDF-15 are a general indication of endothelial and microvascular damage. This may explain the association between GDF-15 and DR since DR is primarily caused by microvascular injury during diabetes (30).

The mechanism of the microvascular effects of GDF-15 on DR in patients with type 2 diabetes has not been elucidated yet. Previous studies have shown that DR is characterized by a decrease in retinal perfusion caused by the constriction of arterioles, endothelial cell degeneration, microvascular destabilization caused by the retinal pericyte loss, and a release of proangiogenic factors that promote the development of many abnormal new vessels (26, 30). In addition, GDF-15 plays a major role in regulating the recruitment of inflammatory cells by directly interfering with leukocyte integrin activation and inhibiting leukocyte arrest and extravasation on the endothelium (30). Due to the anti-inflammatory role of GDF-15 in the vascular endothelial cell, the plasma levels of GDF-15 tend to rise with the progress of microvascular injury (31).

Alternatively, as a member of the TGF-*β* family, GDF-15 may signal through an alternate, non-TGF-*β* receptor mediated mechanism instead of the classical TGF-*β* signaling pathway through Smad phosphorylation (32–34). It may also play an important role in angiogenesis (34). GDF-15 impairs *in vitro* angiogenesis by blocking connective tissue growth factor 2 (CCN-2)-mediated tube formation in human umbilical vein endothelial (HUVEC) cells as well as inhibiting the CCN-2-dependent activation of focal adhesion kinase and subsequent decrease in *α*_V_ *β*_3_ integrin clustering. In the same vein, GDF-15, in combination with BMP-2, has been shown to mediate the inhibition of fenretidine-dependent tumor vessel growth by interfering with endothelial cell growth, migration, and invasion (35). There have been reports that angiogenesis in hypoxic HUVEC cells is promoted by a signaling pathway, which includes hypoxia-inducible factor 1-alpha (HIF-1alpha), VEGF, and p53 (36). Moreover, GDF-15 regulates endothelial cells through altered endothelial caveolar signaling (37). Increased circulating levels of GDF-15 have been attributed, inter alia, to endothelial dysfunction (38). It is possible that the increase in the plasma levels of GDF-15 in the patients with diabetes may cause a counter-regulatory and compensatory mechanism which protects against angiogenesis, but not sufficient to protect against DR. Interestingly, consistent with our hypothesis, we observed that the incidence of DR increased with the plasma GDF-15 quartiles in this study.

This study establishes the association between DR and GDF-15 in type 2 diabetes. There are several limitations to this study. First, due to the cross-sectional nature of the present study, we could not determine whether GDF-15 plays a causal role in the pathogenesis of DR. Accordingly, prospective studies in the future are of vital importance. Second, plasma GDF-15 levels may vary among different populations, and it is unclear how local and ocular factors can influence DR. Third, the sample size was relatively small. Further studies are also required to determine the GDF-15 levels in a larger population. Moreover, VEGF levels in patients with type 2 diabetes may be associated with the development of DR. However, due to the limitations in the study design, we did not measure VEGF levels in the patients. Lastly, given the limited number of study participants with PDR (n = 2), we did not analyze the differences in GDF-15 levels between PDR and NPDR. Based on a previous study (39), PDR was combined with severe NPDR in our study to give the vision-threatening DR (VTDR) group. There is therefore need to investigate the differences between PDR and NPDR.

In conclusion, the results from our study suggest that there is a significant and independent association between the increased plasma levels of GDF-15 and DR. Future prospective studies with greater numbers of patients should be done to provide a link between increased circulating plasma GDF-15 concentration and the severity of DR.

## Data Availability Statement

The raw data supporting the conclusions of this article will be made available by the authors, without undue reservation.

## Ethics Statement

The studies involving human participants were reviewed and approved by the Xinhua Hospital Ethics Committee Affiliated to Shanghai Jiaotong University School of Medicine. The patients/participants provided their written informed consent to participate in this study.

## Author Contributions

Conceived and designed the experiments: ZY and QS. Analyzed the data: YN, WZ, and JS. Contributed reagents/materials/analysis tools: YN, ZY, JS, YL, XL, NL, HZ, and LQ. Wrote the paper: YN, WZ, and JS. All authors contributed to the article and approved the submitted version.

## Funding

This work was supported by grants from National Key R&D Program of China(2016YFC0901200, 2016YFC0901203), the Shanghai Sailing Program (18YF1415800), the Shanghai Science and Technology Commission (15411953200, 10411956600, 14ZR1427400), National Natural Science Foundation of China (81300667, 81370953, 81370935, 81670743), Shanghai Health System Outstanding Young Talents Training Program (XYQ2013098), Shanghai Education Committee Key Program (14zz110), and State Key Development Program for Basic Research of China (2012CB517501).

## Conflict of Interest

The authors declare that the research was conducted in the absence of any commercial or financial relationships that could be construed as a potential conflict of interest.
